# Genomic and Functional Diversity of *Pseudoalteromonas* Associated with the Tropical Bivalve *Anadara tuberculosa*

**DOI:** 10.1007/s00248-026-02753-y

**Published:** 2026-04-24

**Authors:** Mariana Restrepo-Benavides, Pedro Jiménez, María José Figueras, Silvia Restrepo, María Mercedes Zambrano, Isabel Pujol, Marcela Guevara-Suarez, Ana Fernández-Bravo

**Affiliations:** 1 Faculty of Medicine and Health Sciences, Rovira i Virgili University, Tarragona, Spain; 2 Departamento de Ingeniería Química y de Alimentos, Laboratorio de Micología y Fitopatología, Universidad de los Andes, Bogotá, Colombia; 3 Laboratory of Phytopathology, Faculty of Basic and Applied Sciences, Universidad Militar Nueva Granada, Bogotá, Colombia; 4 Boyce Thompson Institute for Plant Research, Ithaca, NY USA; 5 Corpogen (Corporación Corpogen), Bogotá, Colombia; 6 Clinical Laboratory, Hospital Universitari de Sant Joan, Rovira i Virgili University, Reus, Spain; 7 Applied Genomics Research Group, Universidad de los Andes, Bogotá, Colombia

**Keywords:** Marine bacteria, phylogenomics, functional traits, biosynthetic gene clusters, bivalve microbiome, Colombian Pacific

## Abstract

Members of the genus *Pseudoalteromonas* are ecologically versatile marine bacteria involved in nutrient cycling, chemical signaling, and competition for resources via bioactive compound production. Here, we characterize the genomic and functional diversity of 19 *Pseudoalteromonas* isolates from the bivalve *Anadara tuberculosa* to elucidate ecological strategies enabling coexistence in host-associated environments. Whole-genome sequencing and phylogenomic inference revealed extensive genomic diversity, with most isolates affiliated with recognized taxa and two genomically distinct lineages representing candidate novel species. Functional profiling with Traitar identified 67 traits related to enzymatic activity, substrate utilization, and environmental tolerance, revealing a bimodal structure: one assemblage (*Pseudoalteromonas maricaloris*, *Pseudoalteromonas xiamenensis*, *Pseudoalteromonas piscicida*) exhibited diverse biosynthetic pathways consistent with chemical competition strategies, while a second assemblage including the two candidate species displayed broader carbohydrate utilization with streamlined biosynthetic capacity. Notably, unique non-NRPS siderophore clusters in *Pseudoalteromonas gelatinilytica* and one candidate species suggest alternative iron acquisition strategies adapted to host-associated niches. Principal coordinate analysis revealed two distinct ecological assemblages, indicating that functional differentiation through niche partitioning rather than direct competition enables coexistence of phylogenetically related populations within the bivalve microbiome. Comparative genomic metrics (ANI, AAI, dDDH) support the proposal of *Pseudoalteromonas pianguae* sp. nov. and *Pseudoalteromonas iscuandensis* sp. nov., expanding the known diversity of this ecologically important genus. This genome-informed approach provides mechanistic insight into bacterial diversification in tropical bivalve holobionts and establishes a foundation for exploring *Pseudoalteromonas*-mediated interactions in marine invertebrate microbiomes.

**Supplementary Information:**

The online version contains supplementary material available at 10.1007/s00248-026-02753-y.

## Introduction

The genus *Pseudoalteromonas* was first defined in 1995 with bacteria previously included in the genus *Alteromonas* and encompasses Gram-negative, aerobic, motile heterotrophic bacteria within the Gammaproteobacteria class, that are broadly distributed in marine environments where they play a significant ecological role [1, 2]. The genus currently includes 52 validly published species among 69 total entries at the LPSN-List of Prokaryotic names with Standing in Nomenclature (https://lpsn.dsmz.de/genus/pseudoalteromonas as of March 2025). Species of this genus are widely recognized for their metabolic versatility, particularly the production of bioactive compounds such as enzymes, exopolysaccharides, and antimicrobial metabolites, which render them subjects of biotechnological interest [3–5]. Moreover, several *Pseudoalteromonas* species have been identified as key members of marine holobionts, where they may contribute to host development, immune modulation, and defense against pathogens through the secretion of bioactive secondary metabolites. These functional capacities contribute to their ecological success across diverse marine niches, including associations with corals, squids, echinoderms, and bivalves [6–8].

Recent advances in phylogenomic analyses have reshaped our understanding of species delineation within *Pseudoalteromonas*, highlighting the genomic plasticity and taxonomic complexity of this genus [2]. The implementation of high-throughput genome sequencing has facilitated more precise taxonomic resolution using genomic indices such as average nucleotide identity (ANI), digital DNA-DNA hybridization (dDDH), and average amino acid identity (AAI), significantly improving species definition and discrimination [9–11]. The 16 S rRNA gene similarity among *Pseudoalteromonas* species often masks deep genomic divergence, making genome-scale comparisons essential for accurate taxonomic assignments [7].

Previous studies have shown the significant metabolic versatility and ecological adaptability within the genus *Pseudoalteromonas*, including polysaccharide production, biofilm formation, and degradation of complex marine polymers [3–5]. Several species also produce lineage-specific secondary metabolites such as bromoalterochromides and antifouling compounds which have potential biomedical applications [6, 7]. These functional traits underscore the ecological relevance of *Pseudoalteromonas* in marine ecosystems and are consistent with the genomic and phenotypic diversity documented for the genus [2, 6, 8]. However, the diversity and incidence of this bacterial group in tropical environments and in association with mangrove bivalves remain largely unexplored. In particular, filter-feeding bivalves such as *Anadara tuberculosa* concentrate microorganisms from distinct tropical estuarine niches, making them a potential reservoir for unexplored bacterial lineages with unique biosynthetic traits.

Although no culture-independent microbiome study of *A. tuberculosa* has been published to date, *Pseudoalteromonas* has been consistently identified as a core genus in the microbiomes of other bivalve species. In *Mytilus coruscus*, *Pseudoalteromonas* is one of eleven core genera that remains dominant throughout the host life cycle [12] and represents the most abundant genus in the gut microbiota of fast-growing individuals [13]. In *Crassostrea gigas* and *Mytilus galloprovincialis*, *Pseudoalteromonas* is among the dominant genera in hemolymph and digestive gland tissues [14], while in subantarctic mussels (*Mytilus platensis*) it was detected at high abundance in hemolymph at specific sampling sites [15]. These observations suggest that *Pseudoalteromonas* plays an ecologically relevant role in bivalve holobionts, although its relative abundance in *A. tuberculosa* remains to be determined through culture-independent approaches.

Given the importance of accurately describing microbial diversity, the application of integrated polyphasic taxonomic approaches combining phenotypic and genomic analyses remains essential for identifying and characterizing novel marine bacteria. Here, we applied such an approach to characterize *Pseudoalteromonas* strains isolated from the marine bivalve *Anadara tuberculosa*, assigning 17 isolates to five previously described species, delineating two novel taxa, and supporting the reclassification of *P. agarivorans* as a later heterotypic synonym of *P. atlantica.* Collectively, these findings expand our understanding of host-associated bacterial diversity in *A. tuberculosa* and shed light on the ecological breadth of *Pseudoalteromonas* populations inhabiting tropical marine environments.

## Materials and Methods

### Isolation and Preliminary Identification of Bacterial Strains

The bacterial isolates investigated in this study (*n* = 19) were obtained as previously described by Restrepo-Benavides et al. [16]. In brief, specimens of the bivalve *A. tuberculosa* (*n* = 14 in total) were collected from seven sampling sites along the Colombian Pacific coast, three in Buenaventura (Valle del Cauca UTM 18 N) and four in Santa Bárbara de Iscuandé (Nariño UTM 17 N), with two specimens per site (Table S1). Tissue samples were homogenized and plated on nutrient agar (Oxoid, UK) supplemented with seawater filtered through 0.22 μm membrane filters, selecting colonies with distinct morphological characteristics for purification and further analysis.

Preliminary taxonomic identification was performed by amplifying the 16 S rRNA gene directly from bacterial colonies using colony PCR with primers 27 F (5′-AGAGTTTGATCCTGGCTCAG-3′) and 1492R (5′-GGTTACCTTGTTACGACTT-3′). Full details on isolation protocols and thermal cycling conditions are provided in Restrepo-Benavides et al. [16]. Amplicons were sequenced via Sanger technology, and raw sequences were curated and assembled using Geneious Prime v2023.0.4. Taxonomic affiliations were assigned based on sequence similarity searches (BLASTn) against the NCBI nucleotide database using default parameters. In agreement with established thresholds, strains showing ≥ 95% sequence identity in 16 S rRNA were considered to belong to the genus *Pseudoalteromonas* [17], consistent with the phylogenetic framework that originally defined this genus [1]. Selected isolates were stored in nutrient broth containing 15% (v/v) glycerol at -80 °C and routinely cultured on marine agar (Difco, USA) for optimal growth and maintenance.

### Genome Sequencing, Assembly, and Annotation

Genomic DNA from the 19 *Pseudoalteromonas* isolates was extracted using the DNeasy Blood & Tissue Kit (Qiagen, Germany). DNA concentration and integrity were assessed using a Qubit 4.0 Fluorometer (Thermo Fisher Scientific, USA) and 1% (w/v) agarose gel electrophoresis. Whole-genome sequencing was conducted using the Oxford Nanopore Technologies (ONT) platform at the GenCore Sequencing Facility (Universidad de los Andes, Bogotá, Colombia). Barcoding was carried out with the Native Barcoding Expansion 1–96 kit (EXP-NBD196), and libraries were prepared using the Ligation Sequencing Kit (SQK-LSK109), following the manufacturer’s instructions. Sequencing was conducted on a GridION system (ONT, UK) with R9.4.1 flow cells (FLO-MIN106D), and basecalling was carried out with Guppy v6.4.6 in Super Accuracy mode to ensure high-quality reads. Sequencing depth was approximately 100× per genome.

De novo genome assemblies were generated with Flye v2.9.3 [18] using default parameters. Assembly quality was evaluated using QUAST v5.0.2 [19] and genome completeness was evaluated with BUSCO v5.8.2 [20] using the pseudoalteromonas_odb12 lineage dataset and additionally assessed with CheckM v1.2.1 [21] using the lineage-specific workflow. Structural gene annotation was done using Prokka v1.14.6 [22] with default settings. Functional traits were inferred using Traitar [23]. Principal coordinate analysis (PCoA) based on the Jaccard distance of Traitar-predicted traits was performed in R to explore functional clustering among strains. Biosynthetic gene clusters (BGCs) were identified using antiSMASH v7.0 [24] with default strictness, enabling additional analysis modules (ClusterBlast, SubClusterBlast, ActiveSiteFinder). To assess the similarity of identified BGCs to characterized compounds, a gene cluster family (GCF) analysis was performed using BiG-SCAPE v1.1.9 [25] with default parameters (glocal alignment mode, distance cutoff 0.30). The 151 BGCs predicted by antiSMASH were compared against the MIBiG reference database v3.1 [26] to identify gene cluster families with matches to experimentally characterized biosynthetic pathways. Pfam domain annotations (Pfam-A) were used for sequence similarity calculation. Putative plasmid contigs were identified using Plasmer [27] under default configuration.

### Phylogenetic Analyses

Phylogenetic relationships were assessed using three complementary methods: 16 S rRNA gene phylogeny, multilocus phylogenetic analysis (MLPA) using six housekeeping genes, and phylogenomic marker-gene analysis based on 371 conserved single-copy protein-coding markers, as described below. For the 16 S rRNA gene analysis, sequences of 1422 bp from isolates and reference type strains were aligned with ClustalW [28], and neighbor-joining phylogenetic trees were constructed in MEGA v11 [29], employing 1,000 bootstrap replicates. All 52 validly named *Pseudoalteromonas* species were surveyed (LPSN, March 2025). Because genomes for *Pseudoalteromonas aestuariivivens* and *Pseudoalteromonas antarctica* are unavailable, these taxa were included only in the 16 S tree; MLPA and marker-gene phylogenies were restricted to the remaining 50 species with available genomes (Table S1).

The MLPA involved concatenated sequences from six housekeeping genes (*atpD*, *gyrB*, *mreB*, *recA*, *rpoD* and *topA*), genes were retrieved from reference genomes (SAMtools v1.16.1) [30], aligned with ClustalW, concatenated, and inferred by ML under GTR + G+I with 1,000 bootstraps in MEGA v11. Marker-gene phylogeny was inferred from a concatenated alignment of 371 conserved single-copy protein-coding markers identified with PhyloPhlAn v3.1.68 [31]. Homology searches were performed with DIAMOND v2.1.11 [32]; per-marker multiple-sequence alignments were built with MAFFT v7.525 [33] and concatenated. Tree inference used maximum-likelihood in IQ-TREE v2.4.0 under the Q.pfam + F+G4 model with 1,000 ultrafast bootstrap replicates [34]. Resulting trees were visualized with iTOL [35].

### Genomic Relatedness and Species Delineation

Genome-based relatedness metrics were computed for species delineation. ANI was estimated with JSpeciesWS (ANIb/ANIm) [36], OrthoANI v1.2 [37] and FastANI v1.33 [38]; gANI with ANIcalculator v1 [39]; AAI with EzAAI v1.2.1; and dDDH and G + C differences with GGDC v3.0 [10]. Species boundaries were defined a priori as ANI ≥ 95–96%, dDDH ≥ 70%, AAI ≥ 95%, and ΔGC ≤ 1% [9, 39].

### Phenotypic Characterization of Novel Species

Phenotypic characteristics were assessed using standard microbiological procedures [40]. Colony morphology was recorded after growth on marine agar (Difco 2216) at 28 °C for 48 h under aerobic conditions (color, texture, surface, margin). Gram-stain reactions using Hucker’s method and cell morphology were examined by light microscopy. Biochemical profiles were obtained with the Vitek 2 GN system (bioMérieux; 47 enzymatic/assimilation tests) from fresh cultures prepared as above and incubated at 28 °C for 18–24 h per manufacturer’s instructions. Key traits were verified manually: catalase (3% (v/v) H₂O₂), oxidase (Kovács), and starch hydrolysis on marine agar with soluble starch revealed with Lugol’s iodine. Ultrastructure was examined by TEM [41]; images were acquired on a JEOL JEM-1011 at 80 kV.

## Results and Discussion

### Genomic Characteristics of *Pseudoalteromonas* Strains Isolated from *Anadara tuberculosa*

A total of 19 genomes of *Pseudoalteromonas* strains isolated from *A. tuberculosa* were sequenced, including 12 strains from mollusks collected in Buenaventura and seven from Iscuandé that are two mangrove estuarine sites on the Colombian Pacific coast (Table S1). Genome sizes ranged from 4.12 to 5.54 Mb (mean: 4.82 Mb), consistent with previously reported values for the genus [2, 42]. Genome assemblies ranged from two to four contigs (median = 2), with N50 values ranging from 2.64 to 4.23 Mb. Members of the genus *Pseudoalteromonas* characteristically possess bipartite genomes composed of a primary chromosome and a chromid of plasmid origin [43]. The predominance of two-contig assemblies in our dataset is consistent with this multipartite genome architecture, and CheckM completeness values (97.74–100%; mean 99.2%) further support that most assemblies recovered near-complete replicons. The G + C content ranged from 41.1% to 45.2%, in accordance with genomic characteristics typical of *Pseudoalteromonas* [1, 2]. The BUSCO analysis (pseudoalteromonas_odb12 dataset) revealed completeness values between 85.8% and 99.1% (mean: 93.7%), supporting the overall high quality of the assemblies. Single-copy BUSCO scores were similarly high (mean: 92.4%). Fragmentation levels were below 5% in the majority of assemblies, and exceeded this value in only four genomes, reaching a maximum of 9.7%. Gene prediction identified between 3,773 and 5,176 coding sequences (CDS) per genome (median 4,373; mean 4,459 ± 409; *n* = 19), consistent with values reported for other *Pseudoalteromonas* species [8]. No plasmids were detected in any of the assemblies, as none contained contigs classified as plasmid-derived by Plasmer. All genomes consisted exclusively of chromosomal sequences (Table S2).

### Species Delineation Through Phylogenomic Inference and Genome-Wide Similarity Metrics

**Fig. 1:**
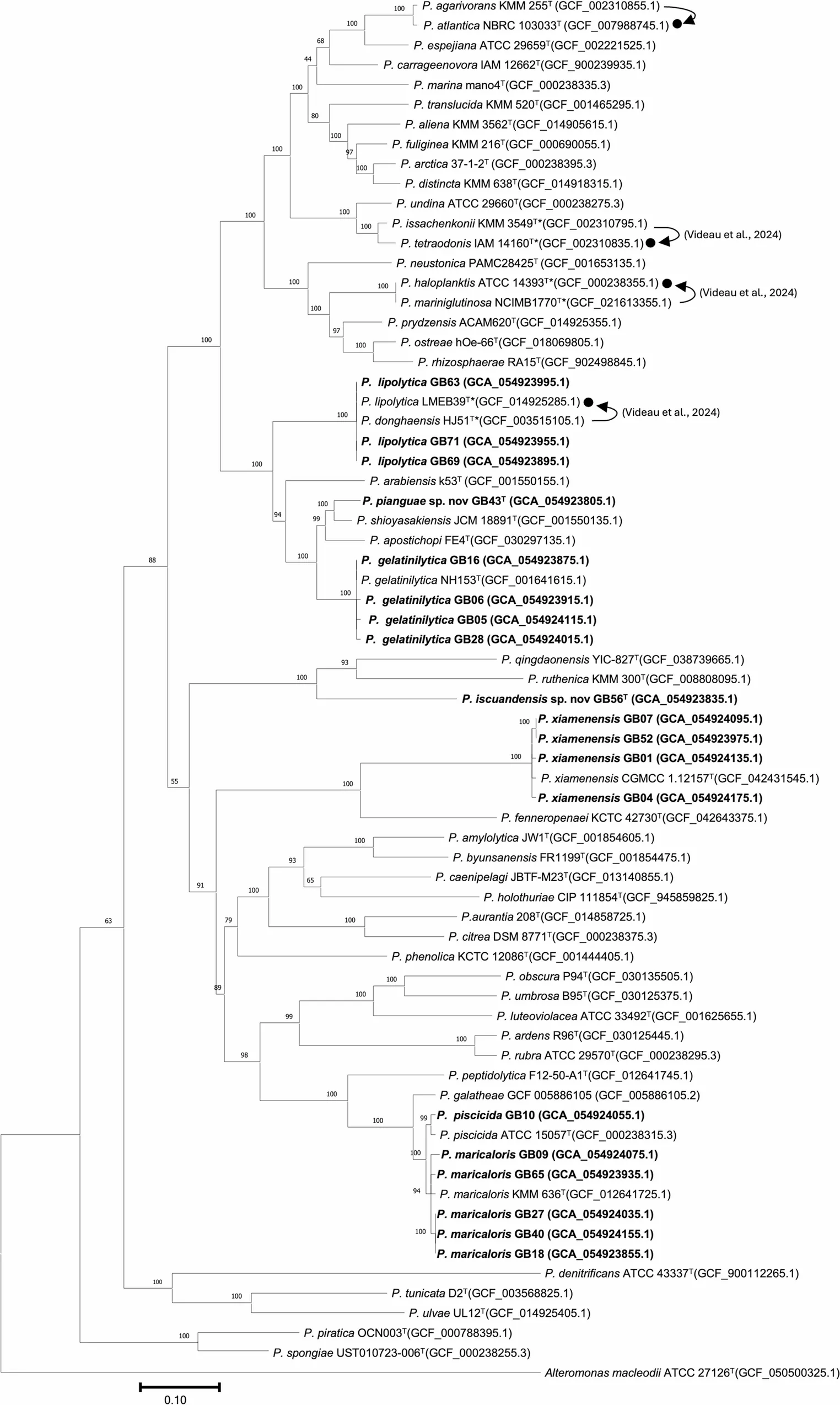
Maximum likelihood phylogenetic tree based on concatenated sequences of six housekeeping genes (*atpD*, *gyrB*, *mreB*, *recA*, *rpoD*, *topA*) from the 19 *Pseudoalteromonas* strains isolated from the bivalve *Anadara tuberculosa* and type strains of the accepted species. Node values indicate bootstrap support (1,000 replicates); only supports ≥ 50% are shown. Bar, substitutions per site. Strains from this study are in bold; type strains marked ^T^. Arrows indicate pairs currently regarded as valid species for which a synonymization is considered; filled circles mark the name that would be retained under the proposed synonymy

**Fig. 2:**
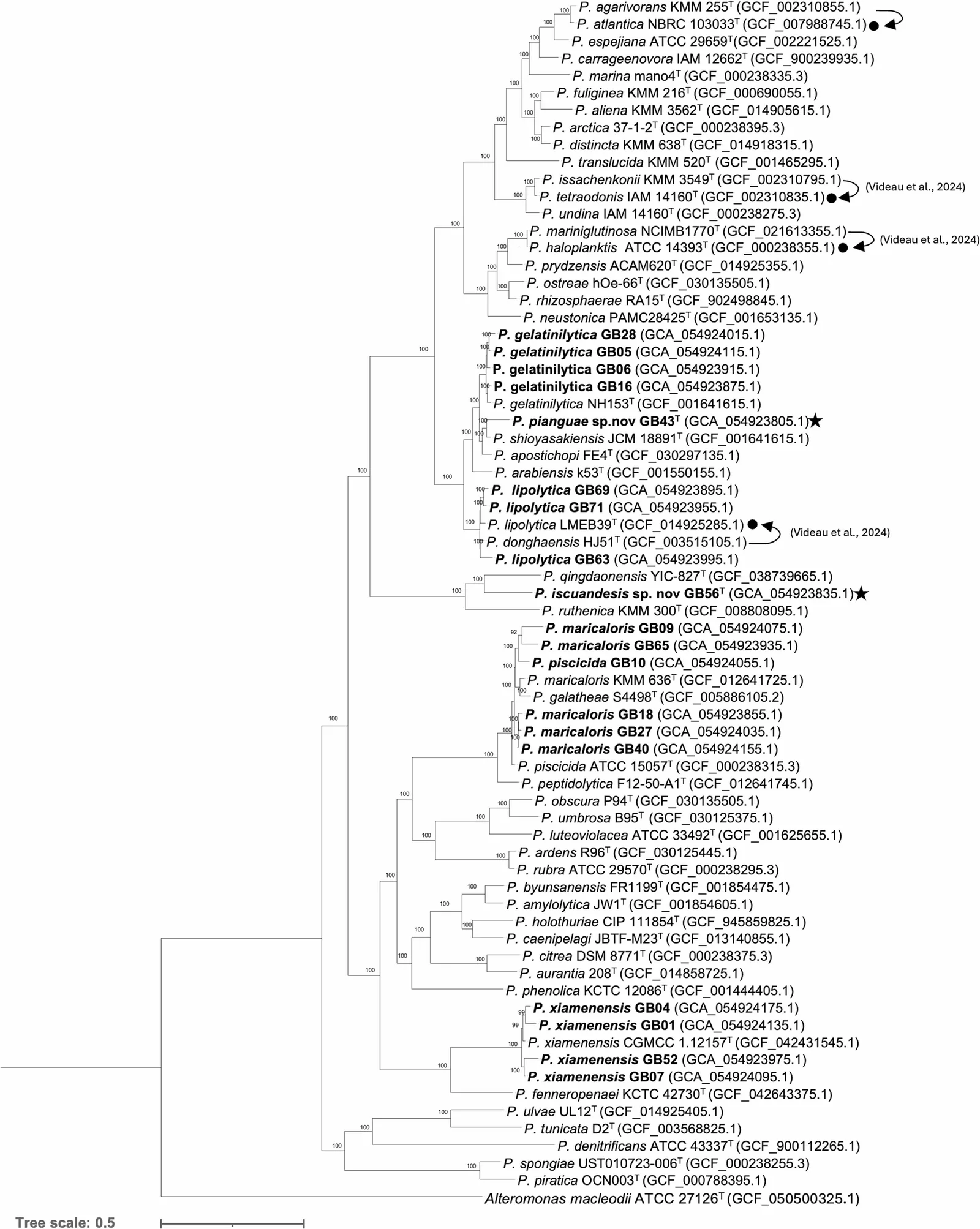
Phylogenomic maximum-likelihood tree inferred from 371 conserved single-copy protein-coding genes across *Pseudoalteromonas* genomes, rooted with *Alteromonas macleodii* ATCC 27126^T^. Node values indicate ultrafast bootstrap support (1,000 replicates); only supports ≥ 50% are shown. Scale bar, substitutions per site. Strains from this study are shown in bold; type strains marked ^T^. Arrows indicate valid-species pairs for which a synonymization is considered; filled circles mark the name that would be retained

To establish the taxonomic position of the 19 *Pseudoalteromonas* isolates, we conducted a multilayered analysis based on 16 S rRNA, multilocus phylogenetic analysis (MLPA), and a phylogenomic marker-gene analysis using a concatenated amino-acid alignment of conserved single-copy proteins. The 16 S rRNA gene-based analysis provided a preliminary assignment, confirming that all isolates belong to the genus *Pseudoalteromonas* by forming a monophyletic clade with type strains (Fig. S1) and showing high similarity to type strains (*≥* 99.07 BLASTn identity). However, due to the limited resolving power of the 16 S rRNA gene at the species level within *Pseudoalteromonas*, 11/19 isolates had multiple, equally close type-strain matches among their top-five BLASTn hits (≥ 99.0% identity; ≥90% query coverage). In contrast, the MLPA of the concatenated sequences of the six housekeeping genes (*atpD*,* gyrB*,* mreB*,* recA*,* rpoD*, and *topA*) provided greater discriminatory power. Seventeen isolates clustered consistently with known species, whereas isolates GB43 and GB56 each formed distinct well-supported lineages with high bootstrap values. In this sense *Pseudoalteromonas shioyasakiensis* was the most closely related species to strain GB43 clustering together with a 100% bootstrap value but formed a distinct lineage, and genome-wide similarity metrics confirmed values well below accepted species-level thresholds (Table 1). Strain GB56 was most closely related to the clade of *Pseudoalteromonas qingdaonensis* and *Pseudoalteromonas ruthenica*, yet formed an independent lineage in all phylogenetic reconstructions, with genome-wide metrics consistently below species-level thresholds (Table 1; Fig. 1). Phylogenomic marker-gene analyses performed with PhyloPhlAn using 371 conserved genes further supported these findings (Fig. 2).

**Table 1:** Genomic relatedness between *Pseudoalteromonas* strains from the bivalve *Anadara tuberculosa* (Genome 2) and their closest phylogenetic relatives (Genome1). Metrics include digital DNA-DNA hybridization (dDDH), Average Nucleotide Identity (ANI), Average Amino Acid Identity (AAI), and differences in genomic G+C content. Genome 1 denotes the reference strain; Genome 2 denotes isolates from this study. The final row compares two type strains, *P. atlantica *NBRC 103033T and *P. agarivorans* KMM 255T

Genome 1	Genome 2	dDDH	ANIb	ANIm	FastANI	gANI	OrthoANI	AAI	G+C (%diff.)
*P. gelatinilytica* NH153^T^	*P. gelatinilytica* GB05	76.50	97.02	97.58	97.26	96.26	97.38	98.32	0.02
	*P. gelatinilytica* GB06	77.90	97.35	97.58	97.42	96.52	97.46	98.44	0.1
	*P. gelatinilytica* GB16	89.30	98.66	98.80	98.74	97.67	98.75	99.14	0.02
	*P. gelatinilytica* GB28	76.80	97.10	97.61	97.37	96.44	97.41	98.23	0.06
*P. lipolytica* LMEB39^T^	*P. lipolytica* GB63	92.70	98.86	99.15	98.97	98.28	99.06	99.17	0.06
	*P. lipolytica* GB69	92.00	98.62	99.97	98.89	98.08	98.99	99.11	0.19
	*P. lipolytica* GB71	92.10	98.61	99.14	98.92	98.07	99.02	99.17	0.18
*P. piscicida* ATCC 15057^T^	*P. piscicida* GB10	74.80	96.61	97.19	96.98	96.51	97.00	97.66	0.05
*P. maricaloris* KMM 636^T^	*P. maricaloris* GB09	77.10	97.07	97.47	97.26	96.77	97.34	98.02	0.08
	*P. maricaloris* GB18	81.50	97.66	97.95	97.86	96.62	97.88	98.49	0.38
	*P. maricaloris* GB27	81.50	97.68	97.96	97.85	96.72	97.95	98.52	0.38
	*P. maricaloris* GB40	81.60	97.72	97.98	97.87	96.73	97.90	98.53	0.39
	*P. maricaloris* GB65	79.10	97.47	97.68	97.47	96.94	97.64	98.29	0.29
*P. xiamenensis* CGMCC 1.12157^T^	*P. xiamenensis *GB01	87.80	98.45	98.64	98.40	98.06	98.59	98.79	0.17
	*P. xiamenensis* GB04	87.50	98.45	98.64	98.39	98.00	98.50	98.85	0.09
	*P. xiamenensis* GB07	86.60	98.34	98.59	98.31	97.90	98.51	98.83	0.19
	*P. xiamenensis* GB52	86.30	98.23	98.56	98.29	97.88	98.42	98.72	0.16
*P. shioyasakiensis* JCM 18891^T^	*Pseudoalteromonas* pianguae sp. nov. GB43^T^	52.00	92.69	94.13	93.83	92.40	93.33	96.02	0.16
*P. apostichopi* FE4^T^		39.70	89.40	90.89	90.45	89.19	90.12	94.47	0.28
*P. qingdaonensis* YIC-827^T^	*Pseudoalteromonas* iscuandensis sp. nov. GB56^T^	20.40	75.57	84.47	80.65	76.86	76.18	82.70	3.76
*P. ruthenica* KMM 300^T^		19.00	72.96	84.19	79.82	74.76	73.80	78.29	2.35
*P. atlantica* NBRC 103033^T^	*P. agarivorans *KMM 255^T^	83.20	97.96	98.26	97.95	97.07	98.12	98.67	0.19

To complement the phylogenetic analyses, we evaluated genomic relatedness using a comprehensive set of whole-genome similarity metrics, including digital DNA-DNA hybridization (dDDH), average nucleotide identity methods (ANIb, ANIm, FastANI, gANI, OrthoANI), average amino acid identity (AAI), and G + C content differences. Comparative analyses were conducted against the closest reference genomes retrieved from GenBank. Seventeen isolates exhibited genomic similarity values consistently above the accepted species-level thresholds across all metrics (dDDH ≥ 70%, ANI ≥ 95–96%, AAI ≥ 95% and G + C difference ≤ 1%) [9, 39], supporting their assignment to previously described species (Table 1).

In contrast, the putative novelties showed genomic similarities below the accepted species-level. Strain GB43 fell well below species cutoffs when compared with the closest species *P. shioyasakiensis* (ANIb 92.69%, dDDH 52.00%) and *Pseudoalteromonas apostichopi* (ANIb 89.40%, dDDH 39.70%). Likewise, GB56 showed very low relatedness to P. qingdaonensis (ANIb 75.57%, dDDH 20.40%) and P. ruthenica (ANIb 72.96%, dDDH 19.00%), all of which are below accepted species thresholds. Taken together with their phylogenetic placement, these genome-scale indices support recognition of GB43 and GB56 as two novel *Pseudoalteromonas* species. Conversely, the pair *P. atlantica* NBRC 103033 and *P. agarivorans* KMM 255 exceeded species-level criteria (ANIb 98.0%, dDDH 83.2%), supporting their treatment as heterotypic synonyms (Table 1). These results, further supported by their clustering in both MLPA and phylogenomic marker-gene analyses, provide robust evidence for the reclassification of *P. agarivorans* as a later heterotypic synonym of *P. atlantica*.

Finally, our genome phylogenomic reconstructions also supported the recent proposals of Videau et al. [2], which recognizes *Pseudoalteromonas mariniglutinosa* as a later heterotypic synonym of *Pseudoalteromonas haloplanktis*, *Pseudoalteromonas donghaensis* as a later heterotypic synonym of *Pseudoalteromonas lipolytica*, and *Pseudoalteromonas issachenkonii* as a later heterotypic synonym of *Pseudoalteromonas tetraodonis*. Taken together, these results support the formal proposal of *Pseudoalteromonas pianguae* sp. nov. (strain GB43^T^ = LMG 34645T) and *Pseudoalteromonas iscuandensis* sp. nov. (strain GB56^T^ = LMG 34646T) as two novel species within the genus. These findings underscore the value of genome-scale approaches in refining bacterial taxonomy and emphasize the importance of the ongoing revisions within *Pseudoalteromonas*.

### Genomic Prediction of Functional and Biosynthetic Potential

**Fig. 3:**
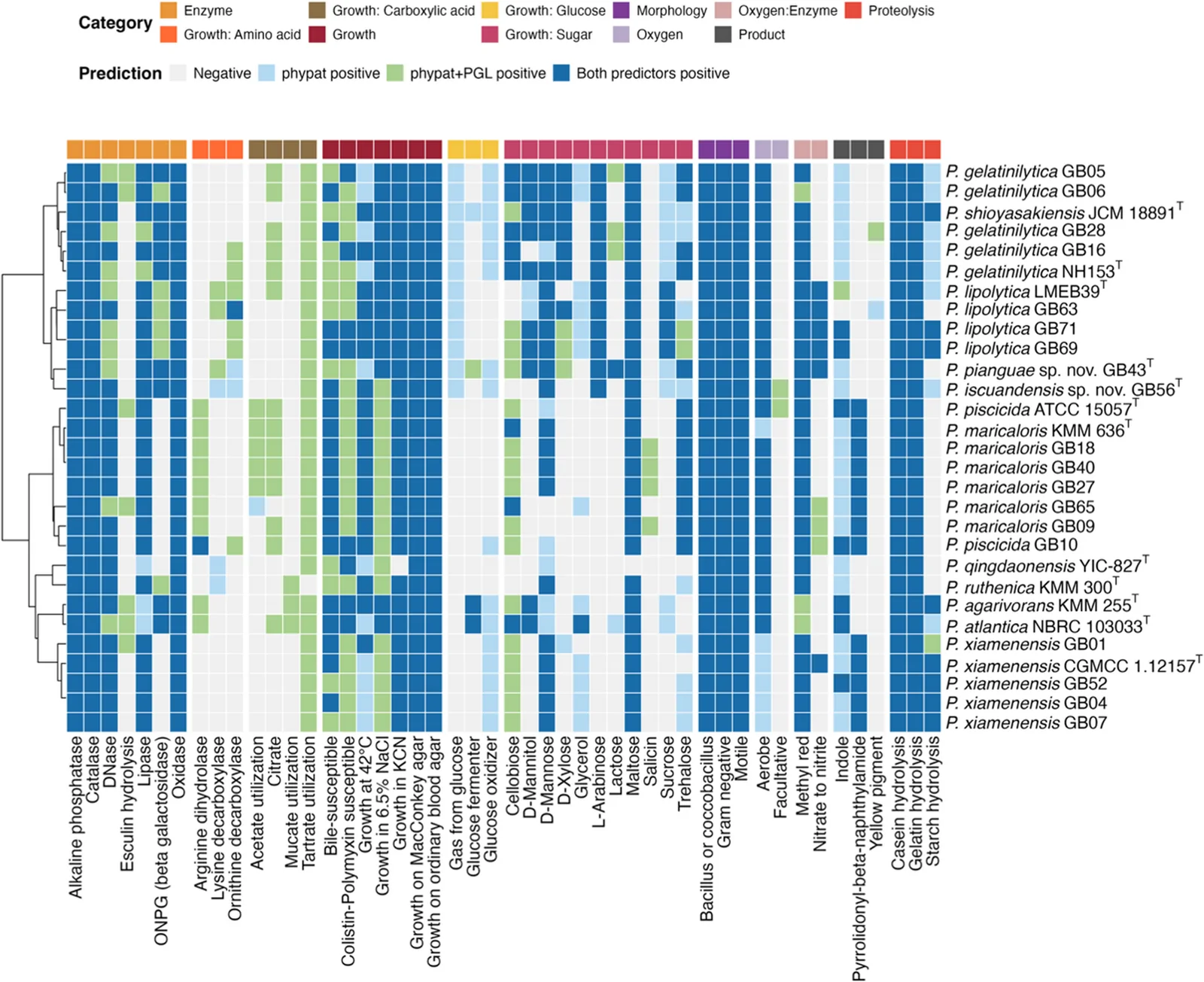
Predicted phenotypic and functional profiles of 19 *Pseudoalteromonas* isolates recovered from the bivalve *Anadara tuberculosa* and ten type strains of *Pseudoalteromonas*, inferred using Traitar. Rows correspond to genomes and columns to predicted traits; traits absent across all genomes were excluded. Columns are grouped by phenotype category (color-coded annotation bar). The dendrogram on the left shows hierarchical clustering of genomes based on Jaccard distance of predicted trait profiles (Ward.D2 method). Prediction status for each trait is indicated in the color key

To explore the functional diversity within the genus *Pseudoalteromonas*, we performed genome-wide phenotype predictions using Traitar software [23], focusing on 19 isolates recovered in this study and ten type strains of validly published species. This comparative framework enabled the identification of lineage-specific traits and broader patterns of metabolic and ecological differentiation. A total of 67 traits were inferred, spanning enzymatic functions, substrate utilization, and environmental tolerances. A core set of 18 traits, including catalase, lipase, DNase, oxidase, casein hydrolysis, motility, and the ability to grow at 42 °C and in 6.5% (w/v) NaCl was conserved across all genomes (Fig. 3). This profile aligns with the known ecological characteristics of the genus, consistent with the genus being Gram-negative, marine-adapted, and proteolytic [44].

Trait-based clustering resolved lineage-linked functional patterns (Fig. 3). Strains affiliated with *Pseudoalteromonas xiamenensis* (GB01, GB04, GB07, GB52 and CGMCC 1.12157^T^) and *P. maricaloris* (GB09, GB18, GB27, GB40, GB65 and KMM 636^T^) shared 26 traits spanning carbohydrate and amino-acid metabolism. *P. maricaloris* was further distinguished by consistent trehalose utilization and predicted arginine dihydrolase activity, as reported in the species description [4]. Although arginine dihydrolase was recovered only by the phypat + PGL model, its uniform presence across all *P. maricaloris* genomes suggests lineage specificity and a role in nitrogen metabolism and pH homeostasis via ammonia production in nutrient-rich, chemically dynamic host microhabitats. Strains assigned to *P. gelatinilytica* (GB05, GB06, GB16, GB28 and NH153^T^) showed broader sugar-fermentation potential (D-xylose, L-arabinose, ONPG/β-galactosidase) including gas from glucose, concordant with phenotypic data [45]. *P. lipolytica* (GB63, GB69, GB71 and LMEB39^T^) encoded utilization of glycerol, mannitol and ornithine plus nitrate reduction, in line with prior reports [46]. Notably, *P. lipolytica* shared L-arabinose utilization, ONPG/β-galactosidase activity, and gas from glucose with *P. gelatinilytica*, although the two lineages differed in D-xylose and cellobiose fermentation, indicating partially overlapping but distinct substrate utilization profiles. The functional profile of GB10 (*P. piscicida*) displayed a functional profile largely shared with other isolates. Relative to the *P. piscicida* type strain ATCC 15057 ^T^, GB10 encoded nitrate reduction and ornithine decarboxylase, while the type strain was distinguished by acetate utilization and esculin hydrolysis, indicating strain-level variation within this species [4] (Fig. 3).

The distribution of enzymatic capacities and substrate use indicates resource partitioning among co-occurring lineages, consistent with niche differentiation in host-associated communities. Metabolically versatile taxa (*P. maricaloris*, *P. xiamenensis*, *P. piscicida*) likely dominate the breakdown of diverse organic substrates within the bivalve microbiome, whereas Group II lineages (*P. gelatinilytica*, *P. lipolytica*, *P. pianguae* sp. nov., *P. iscuandensis* sp. nov.) retain narrower, lineage-specific capacities (e.g., alternative sugars, nitrate reduction) indicative of focused strategies.

Both novel species, *Pseudoalteromonas pianguae* sp. nov. (GB43) and *Pseudoalteromonas iscuandensis* sp. nov. (GB56), exhibited functional profiles aligned with the ecological features of host-associated marine bacteria. Their genomes encoded catalase, oxidase, lipase, alkaline phosphatase, and β-galactosidase activities, along with the predicted ability to utilize carbohydrates such as D-maltose. These capabilities suggest metabolic versatility and adaptation to nutrient-rich microenvironments such as bivalve tissues. In *P. pianguae* sp. nov., additional predicted traits included lactose fermentation and nitrate reduction, which were not observed in its closest phylogenetic relative *P. shioyasakiensis*. Conversely, P. *pianguae* sp. nov. lacked starch hydrolysis capacity, a function present in *P. shioyasakiensis*. These functional differences reinforce the ecological and physiological distinctiveness of *P. pianguae* sp. nov. and support its recognition as a novel species. In the case of *P. iscuandensis* sp. nov., trait predictions indicated the ability to utilize L-arabinose, a function absent in its closest relatives *P. qingdaonensis* and *P. ruthenica* (Fig. 3). These functional differences reinforce the ecological and physiological distinctiveness of both novel species.

To complement these findings and gain further insight into ecological adaptation and potential antimicrobial capabilities, we explored the biosynthetic potential of each lineage through genome mining of secondary metabolite BGCs across the 19 isolates. *P. xiamenensis *strains harbored conserved clusters for prodigiosin (pigmentation and antimicrobial activity), lanthipeptide class I (antimicrobial activity), and PKS-like/butyrolactone biosynthesis [42, 47, 48]. The butyrolactone BGC detected by antiSMASH is classified based on AfsA-family biosynthetic genes, historically linked to γ-butyrolactone signaling in Actinobacteria [49]; although homologs of these genes have been identified across diverse bacterial phyla [50], canonical quorum sensing in *Pseudoalteromonas* is mediated by N-acyl homoserine lactones through LuxI/LuxR-type systems [51], and the functional role of these predicted clusters in our isolates remains to be experimentally determined.

**Fig. 4:**
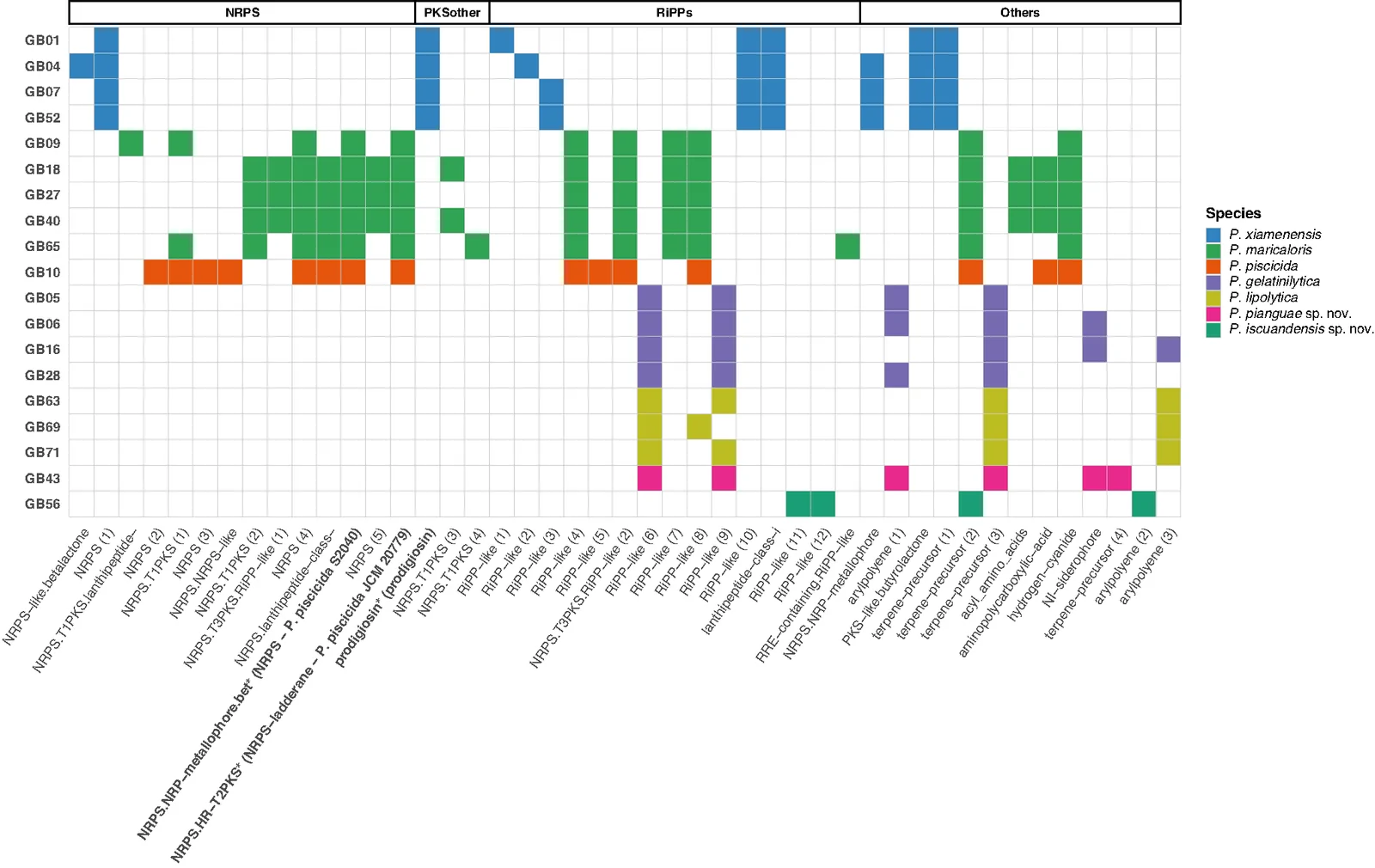
Gene cluster families (GCFs) of biosynthetic gene clusters across 19 *Pseudoalteromonas* isolates from *Anadara tuberculosa*. Rows are genomes grouped by species; columns are GCFs. Filled squares indicate presence. The top annotation bar shows the BiG-SCAPE class assignment for each GCF. Bold column labels denote GCFs with matches to characterized compounds in the MIBiG database

*P. maricaloris* exhibited the highest BGC diversity, with isolate GB65 encoding NRPS, T1PKS, NRP-metallophore, betalactone, lanthipeptide, hydrogen cyanide, and thiopeptide clusters, a BGC class widely distributed across bacterial phyla [52, 53] but not previously reported in *Pseudoalteromonas* genome mining surveys [42] (Fig. 4). This repertoire suggests a chemical warfare strategy centered on antimicrobial production and resource competition. The co-occurrence of thiopeptides (potent antimicrobials) and hydrogen cyanide biosynthetic pathways indicates this lineage employs multifaceted chemical defenses to monopolize nutrient-rich microhabitats within the bivalve microbiome [42, 47, 54]. In contrast, the detection of non-NRPS siderophore clusters specifically in *P. gelatinilytica* and *P. pianguae* sp. nov. represents an alternative iron acquisition strategy, suggesting that distinct iron acquisition mechanisms may contribute to niche differentiation in iron-limited environments among the co-habitant *Pseudoalteromonas* species [55–57].

Strains of *P. gelatinilytica* and *P. lipolytica* exhibited limited BGC repertoires, primarily composed of arylpolyene and RiPP-like clusters. Arylpolyene clusters, associated with pigmentation and protection against oxidative stress, were distributed across several Group II lineages including *P. gelatinilytica* (GB05, GB06, GB16, GB28), *P. lipolytica* (GB63, GB69, GB71), *P. pianguae* sp. nov. (GB43), and *P. iscuandensis* sp. nov. (GB56), but were absent in *P. maricaloris* and *P. xiamenensis*. BiG-SCAPE analysis resolved these arylpolyene clusters into three distinct GCFs, each exclusive to a subset of Group II strains, suggesting lineage-specific diversification within this BGC class (Fig. 4).

Sequence similarity networking of the 151 BGCs identified across all 19 isolates using BiG-SCAPE [25] grouped them into 45 non-redundant gene cluster families (GCFs) at a distance cutoff of 0.30. Comparison against the MIBiG v3.1 reference database of experimentally validated BGCs [26] revealed that only three GCFs (6.7%) had significant similarity to characterized clusters, whereas 42 GCFs (93.3%) lacked any known counterpart. Among the matched families, a PKS-like GCF conserved across all four *P. xiamenensis* strains (GB01, GB04, GB07, GB52) showed similarity to the prodigiosin cluster of *P. rubra* (MIBiG: BGC0002675), consistent with the pigmentation phenotype observed in this lineage. Two additional NRPS-type GCFs were shared by all five *P. maricaloris* isolates and the *P. piscicida* strain GB10, matching previously characterized NRPS clusters from *P. piscicida* (MIBiG: BGC0002491, BGC0000314). Notably, none of the BGCs from the specialized lineages, *P. gelatinilytica*, *P. lipolytica*, *P. pianguae* sp. nov., and *P. iscuandensis* sp. nov., matched any MIBiG entry, suggesting that the biosynthetic potential of these taxa remains entirely uncharacterized. The three arylpolyene clusters detected in these lineages segregated into three distinct GCFs, each exclusive to a subset of Group II strains, while the non-NRPS siderophore clusters of *P. gelatinilytica* (GB06, GB16) and *P. pianguae* sp. nov. (GB43) formed a single GCF with no characterized counterpart. Collectively, these results indicate that the metabolically versatile species harbor BGC repertoires with partial overlap to known chemistry, whereas the specialized lineages represent a largely untapped reservoir of biosynthetic novelty (Fig. 4; Table S4).

**Fig. 5:**
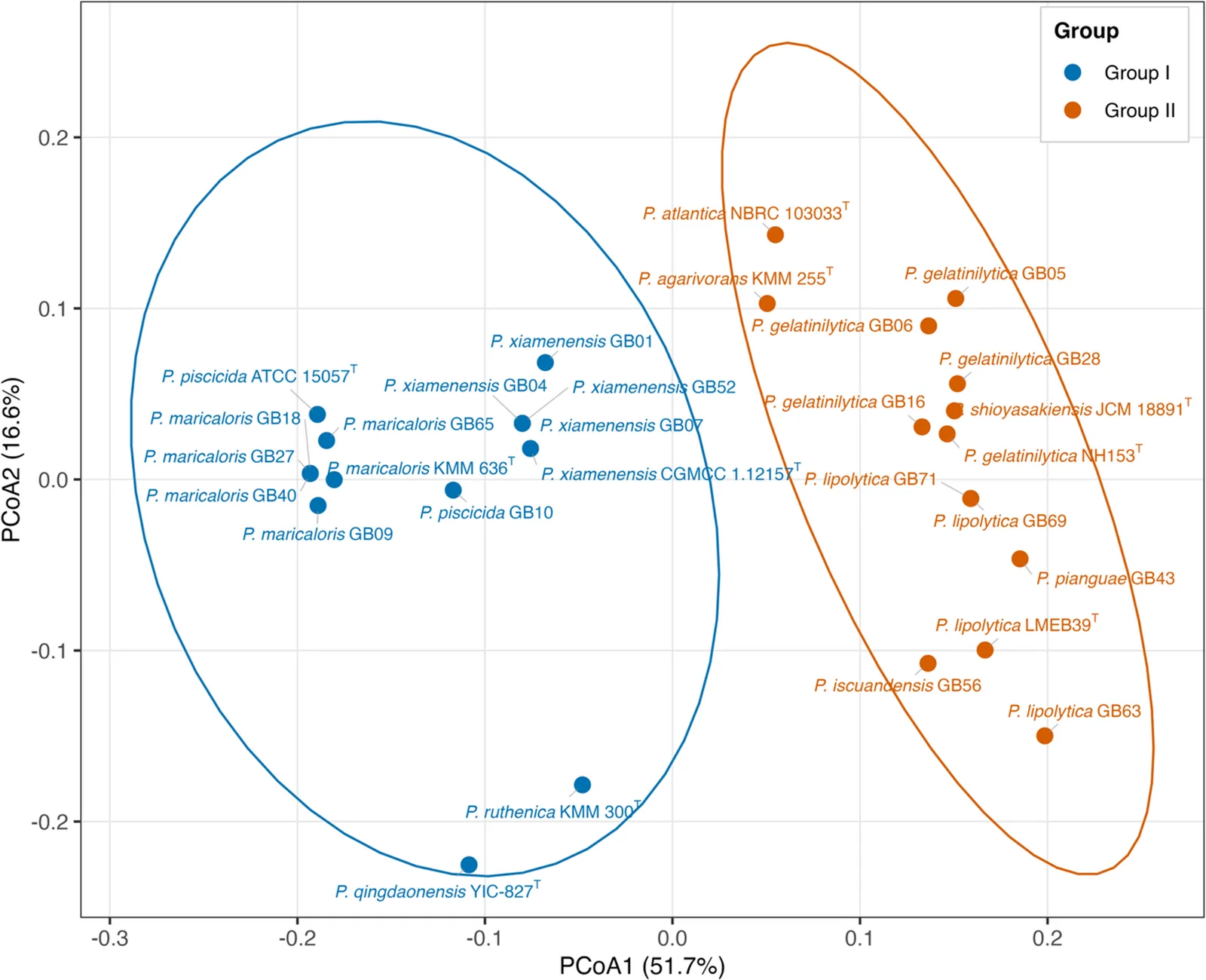
Principal coordinate analysis (PCoA) of functional traits predicted by Traitar for 19 *Pseudoalteromonas* isolates from *Anadara tuberculosa* and ten reference type strains. The ordination is based on Jaccard distances calculated from a binary presence-absence matrix of 67 predicted phenotypic traits. Colors indicate two functional assemblages identified through clustering analysis: Group I (blue; *P. maricaloris*, *P. xiamenensis*,* P. piscicida*, and related reference strains) characterized by diverse biosynthetic profiles, and Group II (orange; *P. gelatinilytica*,* P. lipolytica*,* P. pianguae* sp. nov., *P. iscuandensis* sp. nov., and related reference strains). Ellipses represent 95% normal confidence regions. The first two principal coordinates explain 68.3% of total variance. Statistical support for the functional bipartition is presented in the text

To further assess the functional differentiation inferred from genome-based phenotype predictions, we performed a principal coordinate analysis (PCoA) using the binary presence-absence matrix of Traitar-predicted traits (Fig. 5). The analysis revealed two distinct functional assemblages: Group I (*P. maricaloris*,* P. xiamenensis*,* P. piscicida*) enriched in biosynthetic gene clusters associated with antimicrobial production and chemical competition, and Group II (*P. gelatinilytica*,* P. lipolytica*,* P. pianguae* sp. nov., *P. iscuandensis* sp. nov.) displaying broader carbohydrate utilization capacities but streamlined BGC profiles. The first two principal coordinates explained 68.3% of the total variance (PCoA1 = 51.7%, PCoA2 = 16.6%). This functional bipartition was statistically supported by PERMANOVA (R² = 0.47, F = 23.6, *p* = 0.001) and an average silhouette coefficient of 0.42, indicating that genomic diversity translates into distinct ecological strategies within the genus. Dispersion homogeneity between groups was confirmed by betadisper analysis (F = 0.24, *p* = 0.626), validating the robustness of the observed functional clustering. Group I exhibited functional profiles dominated by diverse biosynthetic pathways and larger genomes (mean: 5.04 ± 0.54 Mb, *n* = 10), whereas Group II displayed broader substrate utilization capacities with fewer secondary metabolite clusters and smaller genomes (mean: 4.65 ± 0.24 Mb, *n* = 9; Welch’s t-test, *p* = 0.06). This functional bipartition suggests that resource partitioning, rather than direct competition, may enable coexistence of phylogenetically related *Pseudoalteromonas* populations within the spatially structured bivalve microbiome. However, the ecological interpretation of these patterns requires caution, as TRAITAR assesses only 67 phenotypic traits and additional functional dimensions remain unexplored. The functional differentiation observed aligns with ecological theory predicting that resource partitioning reduces competitive exclusion in species-rich microbial communities. We acknowledge that the ecological interpretations presented here are based on cultured isolates and that culture-independent approaches would be necessary to establish the relative abundance and metabolic activity of *Pseudoalteromonas* within the *A. tuberculosa* microbiome.

Notably, strains within each functional group may exhibit distinct ecological dynamics. Within Group I, *P. maricaloris* and *P. xiamenensis* share similar TRAITAR profiles yet possess divergent BGC repertoires, suggesting that chemical competition or niche differentiation at the level of secondary metabolism may occur despite overall functional similarity. Conversely, *P. gelatinilytica* and *P. lipolytica* in Group II share both predicted phenotypes and BGC profiles, which could facilitate coexistence through similar resource utilization strategies or, alternatively, intensify competition for overlapping niches. Furthermore, the novel species may appear functionally streamlined partly due to annotation bias, as TRAITAR’s training database may underrepresent lineage-specific genes; future studies employing KEGG or eggNOG annotations could provide additional functional resolution.

Finally, to assess the consistency between genome-based predictions and experimentally determined phenotypes, we compared Traitar results with biochemical profiles obtained from the Vitek 2 GN system for both novel *Pseudoalteromonas* strains (Table S3). Out of ten comparable traits, *P. pianguae* sp. nov. showed full agreement in four traits, hydrogen sulfide production, D-sorbitol utilization, urea hydrolysis, and phosphatase activity whereas *P. iscuandensis* sp. nov. matched in six traits, including the same four and additionally D-mannitol and D-cellobiose utilization. In both strains, discordance was observed for sucrose, ornithine and lysine decarboxylase, and trehalose, where Vitek results were negative and Traitar predicted presence. These discrepancies likely reflect differences in expression regulation or detection thresholds. This highlights the partial overlap between phenotype and genome-based approaches. The lower-than-expected concordance likely reflects the underrepresentation of *Pseudoalteromonas* in TRAITAR’s training database [23] as well as differences between genomic potential and expressed phenotypes under specific assay conditions. These findings underscore the value of integrating both computational and experimental approaches in robust species characterization.

### Phenotypic and Morphological Characterization of Novel Species

Both novel species exhibited phenotypic traits characteristic of *Pseudoalteromonas*, including aerobic metabolism, rod-shaped morphology, motility, and positive catalase and oxidase activities. *P. pianguae* sp. nov. formed pale cream to yellowish colonies on marine agar and was distinguished from *P. apostichopi* and *P. shioyasakiensis* by nitrate reduction capability and growth at both 37 °C and 42 °C, traits absent or restricted in its closest relatives (Table S3, Fig. S3). *P. iscuandensis* sp. nov. produced translucent, pale cream colonies and was differentiated from *P. qingdaonensis* and *P. ruthenica* by negative assimilation of sucrose, D-maltose, and D-mannitol, positive β-N-acetylglucosaminidase activity, and growth at 42 °C exceeding *P. ruthenica*’s upper limit (Table S3, Fig. S3). These phenotypic distinctions, combined with morphological and genomic evidence, provide a comprehensive basis for recognizing both as novel species.

### Description of Pseudoalteromonas Pianguae sp. nov

*Pseudoalteromonas pianguae* (pi.an’gua.e. N.L. gen. fem. n. *pianguae*, from “piangua”, Colombian name for *Anadara tuberculosa*). Cells are Gram-negative, aerobic, motile rods (1.5–2.6 × 0.5–0.9 μm). Colonies on marine agar are pale cream to yellowish, circular, smooth, mucoid after 48 h at 28 °C. Grows at 15–42 °C (optimum 28 °C). Catalase and oxidase positive; reduces nitrate; H₂S negative. Key differential traits: nitrate reduction positive (absent in *P. shioyasakiensis* and *P. apostichopi)*; grows at 37 °C and 42 °C (*P. apostichopi* negative at both). Complete characterization in Table S3 and Fig. S3. DNA G + C 41.12 mol%. Type strain GB43^T^ (=LMG 34645T), isolated from *A. tuberculosa* mantle, Santa Bárbara de Iscuandé, Colombia.

### Description of Pseudoalteromonas Iscuandensis sp. nov

*Pseudoalteromonas iscuandensis* (is.cuan.den’sis. N.L. fem. adj. *iscuandensis*, from Iscuandé, Colombia). Cells are Gram-negative, aerobic, motile rods (1.6–2.3 × 0.4–0.7 μm). Colonies on marine agar are pale cream, circular, translucent, smooth, mucoid after 24 h at 28 °C. Grows at 28–42 °C (optimum 28 °C). Catalase, oxidase, Ala-Phe-Pro arylamidase, L-proline arylamidase, β-N-acetylglucosaminidase, tyrosine arylamidase, and alkaline phosphatase positive. Nitrate reduction, H₂S, β-glucosidase, urease, and glucose fermentation negative. Key differential traits: β-N-acetylglucosaminidase positive (like *P. ruthenica*, unlike *P. qingdaonensis*); grows at 42 °C (exceeds *P. ruthenica* limit); sucrose, D-maltose, D-mannitol negative (*P. qingdaonensis* positive). Complete profile in Table S3 and Fig. S3. DNA G + C 45.18 mol%. Type strain GB56^T^ (=LMG 34646T), isolated from *A. tuberculosa* mantle, Santa Bárbara de Iscuandé, Colombia.

### Emended Description of Pseudoalteromonas Atlantica

The description of *Pseudoalteromonas* atlantica by Gauthier and colleagues (1995) is emended based on genome-wide comparisons and phylogenomic inference. The genome of strain *P. atlantica* NBRC 103033 shares 97.96% ANIb, 98.26% ANIm, 97.95% FastANI, 97.07% gANI, 98.12% OrthoANI, and 98.67% AAI with *Pseudoalteromonas agarivorans* KMM 255 Romanenko et al. 2003. The dDDH value is 83.20%, exceeding the species delineation threshold. The difference in G + C content between the two genomes is 0.19 mol%. These results are further supported by phylogenomic analyses based on MLPA and core-genome trees inferred with PhyloPhlAn, which consistently cluster *P. agarivorans* and *P. atlantica* in a monophyletic clade with 100% bootstrap support (Figs. 1 and 2). These findings confirm that both strains belong to the same species and expand the known genomic and phylogenetic diversity within *P. atlantica*. Based on these results, *Pseudoalteromonas agarivorans* is proposed as a later heterotypic synonym of *Pseudoalteromonas atlantica*.

## Supplementary Information

Below is the link to the electronic supplementary material.

Supplementary Material 1

Supplementary Material 2

## Data Availability

All sequence data are available under BioProject PRJNA1178291; individual accessions are listed in Table S1.
