# Supplementary material for: Genomic and Functional Diversity of Pseudoalteromonas Associated with the Tropical Bivalve Anadara tuberculosa
Source: Microb Ecol. 2026 Apr 24;89:152. doi: 10.1007/s00248-026-02753-y (PMC13433383; doi:10.1007/s00248-026-02753-y)
Supplement: Supplementary file 1 [file 248_2026_2753_MOESM2_ESM.docx]

**Figure S1.** Neighbor-joining phylogenetic tree based on 16S rRNA gene sequences (1422 bp) showing the position of *Pseudoalteromonas* strains isolated from *Anadara tuberculosa* in relation to type strains of the genus *Pseudoalteromonas*. Bootstrap values (>50%) based on 1,000 replicates are shown at branch points. The scale bar indicates substitutions per nucleotide position. Strains isolated in this study are indicated in bold, and type strains are denoted by a superscript T.

**Figure S2.** Distribution of biosynthetic gene cluster (BGC) types predicted by antiSMASH across 19 Pseudoalteromonas isolates from Anadara tuberculosa. Rows are genomes grouped by species; columns are BGC types ordered by parent category. Filled squares indicate presence. The top annotation bar shows the parent category for each BGC type.

**Figure S3.** Transmission electron micrographs of novel *Pseudoalteromonas* species.**A)** *Pseudoalteromonas pianguae* sp. nov. (strain GB43ᵀ). **B)** *Pseudoalteromonas iscuandensis* sp. nov. (strain GB56ᵀ).

A)

B)
